# Long-term effects of SARS-CoV-2 infection on human brain and memory

**DOI:** 10.1038/s41420-023-01512-z

**Published:** 2023-06-29

**Authors:** Qiulu Ding, HanJun Zhao

**Affiliations:** 1 School of Finance and Business, Shanghai Normal University, Shanghai, China; 2 School of Education, Shanghai Normal University, Shanghai, China; 3 Department of Microbiology, School of Clinical Medicine, LKS Faculty of Medicine, The University of Hong Kong, Pokfulam, Hong Kong Special Administrative Region China; 4 Centre for Virology, Vaccinology and Therapeutics, Science Park, Hong Kong Special Administrative Region China

**Keywords:** Cell biology, Immunology

## Abstract

The severe acute respiratory syndrome coronavirus 2 (SARS-CoV-2) variants have caused several waves of outbreaks. From the ancestral strain to Omicron variant, SARS-CoV-2 has evolved with the high transmissibility and increased immune escape against vaccines. Because of the multiple basic amino acids in the S1-S2 junction of spike protein, the widespread distribution of angiotensin-converting enzyme 2 (ACE2) receptor in human body and the high transmissibility, SARS-CoV-2 can infect multiple organs and has led to over 0.7 billion infectious cases. Studies showed that SARS-CoV-2 infection can cause more than 10% patients with the Long-COVID syndrome, including pathological changes in brains. This review mainly provides the molecular foundations for understanding the mechanism of SARS-CoV-2 invading human brain and the molecular basis of SARS-CoV-2 infection interfering with human brain and memory, which are associated with the immune dysfunction, syncytia-induced cell death, the persistence of SARS-CoV-2 infection, microclots and biopsychosocial aspects. We also discuss the strategies for reducing the Long-COVID syndrome. Further studies and analysis of shared researches will allow for further clarity regarding the long-term health consequences.

## Facts

- SARS-CoV-2 can cause Long-COVID syndrome in patients of different age groups.
- SARS-CoV-2 can invade the neural system to interfere with human memory.
- SARS-CoV-2 infection can induce cell death in multiple organs

## Open questions

- What are the mechanisms of SARS-CoV-2 infection causing Long-COVID syndrome?
- What are the risk factors increasing the duration time and severity of Long-COVID syndrome?
- How can we reduce the morbidity rate of Long-COVID syndrome and memory problems caused by SARS-CoV-2 infection?
- What are the roles of biopsychosocial aspects affecting Long-COVID syndrome?

## Introduction

The outbreak of SARS-CoV-2 has generated many variants with the high transmissibility since the emergence of SARS-CoV-2 in 2019. After the outbreak of Omicron variant, there are more than 0.7 billion infectious cases distributed widely in the world. Although many countries have high vaccination rates, the reported cases of the breakthrough infection of the Omicron variant in vaccinated patients are increased. During the past 3 years, an estimated 10–15% of SARS-CoV-2 patients had shown the post-COVID-19 symptoms (namely as Long-COVID syndrome), including fatigue, hyposmia shortness of breath, headache, smell or taste disturbance, myalgia, anxiety, and brain frog (memory impairment and other cognitive symptoms) [1–5]. These symptoms were more common in patients with severe clinical courses [2]. To make the definition of ‘post COVID-19 condition’ clear, the WHO clarified that in addition to a period of usually 3 months from the symptom onset, the presence of at least one symptom (such as fatigue, shortness of breath and others) should last for more than two months. The incidence of dementia and psychiatric disorders in patients with SARS-CoV-2 infection was significantly higher than that in patients infected with influenza virus or other respiratory viruses [6]. The subclinical cognitive impairment was also a common complication after the recovery from mild to moderate COVID-19 in some young patients [4]. In addition, encephalopathy during the acute phase of COVID-19 might be the important risk factor to patients and the prognosis was more related to neuronal damage [7]. Thus, the Long-COVID syndrome may unmask the potential risks for both of patients with severe infectious courses and patients with mild symptoms. In this review, we talk about the molecular evidences of SARS-CoV-2 infection in brains and the effects of spike-mediated syncytia on brains. We further illustrate the possible long-term effects of SARS-CoV-2 infection on human brain and memory, the mechanisms and factors of SARS-CoV-2 affecting human brain and memory. Finally, we discuss the strategies for preventing the Long-COVID syndrome, which may provide the useful information to combat SARS-CoV-2 infection for our daily life.

## The possible routes of SARS-CoV-2 invading human brains

**Fig. 1:**
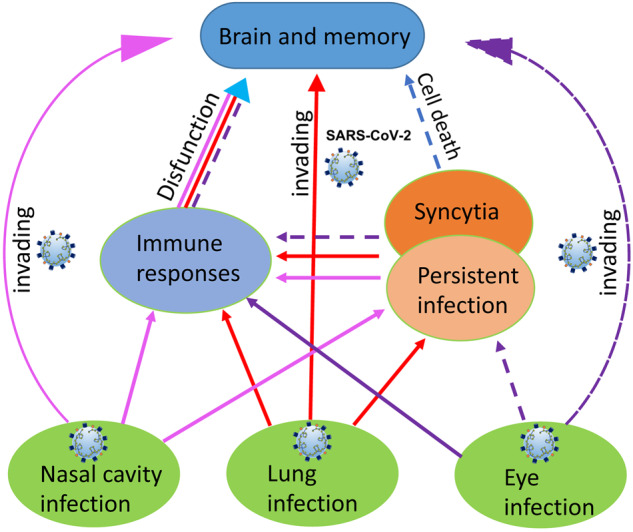
The schematic map of SARS-CoV-2 infecting human brain and affecting human memory. SARS-CoV-2 could directly infect the nasal cavity, lungs and eyes. High viral titers could be detected in the nasal cavity and lungs, which are likely to cause the direct viral invasion to brains through olfactory sensory neurons and the blood–brain barrier. The efficient viral replication in multiple organs can cause the exposure of these organs to high titers of SARS-CoV-2 and stimulate host immune responses and neuroinflammation to induce neurological symptoms. Persistent viral replication and syncytia formation can stimulate the production of cytokines and autoantibodies to affect the brain and memory with the long-term dysfunction. The eye infection with low viral replication is a potential route to affect the brain and memory. Color lines indicate the corresponding routes. Solid lines indicate the direct effects and dot lines indicate the routes of the potential effects.

The respiratory tract is the primary organ infected with SARS-CoV-2. How can SARS-CoV-2 infect the brain after SARS-CoV-2 replication in the respiratory tract? At least three routes of SARS-CoV-2 invading brains are possible. First, SARS-CoV-2 infection in the nasal cavity could lead the virus to directly spread to the olfactory bulb of the brain through the olfactory nerves [8, 9]. The high concentration of the ACE2 receptor expressed in nasal cavity allows the nasal surface cells to serve as the dominant infectious site for SARS-CoV-2. In this condition, the olfactory sensory neurons are fully exposed to SARS-CoV-2, which provides the direct route for neuroinvasion. The detection of SARS-CoV-2 RNA and viral proteins in the brain regions indicated the infection of SARS-CoV-2 in brains [9]. Second, the viral infection in the respiratory tracts with high viral titers could cause significant pathology changes, which can allow the virus to invade the blood system and then virus can spread to the multiple organs with the expression of cell membrane ACE2 or the soluble ACE2 expression [9]. The central nervous system (CNS) of the brain is carefully sheltered by the blood–brain barrier. Before viral invasion into the brain, viral pathogens need to penetrate the blood–brain barrier [10, 11]. When SARS-CoV-2 was traveled to brains, the entry of SARS-CoV-2 into CNS could happen via different pathways, such as ACE2-mediated transcellular pathway, the paracellular pathway by disrupting the tight-junctions of the blood–brain barrier, and the intracellular cargo in which viral particles inside a cell can be transported to CNS by circumventing the blood–brain barrier [8, 12]. Third, SARS-CoV-2 could infect eyes and then reach the occipital cortex through the optic nerve [13–15]. Although eyes were not the main infectious organ with low viral loads [16, 17] and some COVID-19 cases with conjunctivitis as the only symptom were reported [18–20], this route is possible for brain infection [21, 22]. Overall, these routes and entry pathways provide the different chances of SARS-CoV-2 invading brains (Fig. 1).

## ACE2 expression in human brain supporting SARS-CoV-2 replication

ACE2 is the key receptor for SARS-CoV-2 entry into host cells and viral replication in cells (Table 1). The one important factor to affect SARS-CoV-2 replication is the receptor ACE2 distribution in the different organs. Studies showed that ACE2 could be detected in the brain although various studies demonstrated different expression levels [23, 24]. Human brain organoid and tissue studies showed that ACE2 could be detected in the mature choroid plexus cells [25], the cortical gray matter [23] and the olfactory bulb areas of human brain tissues [26]. Further studies demonstrated that ACE2 antibody could block SARS-CoV-2 infection in human brain organoids [23]. Moreover, human brain organoid studies showed that SARS-CoV-2 could replicate in human brain organoids with increased viral titers [27]. These studies showing ACE2 expression in human brains provided the molecular basis and evidence of SARS-CoV-2 infection and replication in brains.

**Table 1:** The host receptors for SARS-CoV-2 infection based on current data.

Receptors	Cells and organs expressing the receptors	Reference
Angiotensin-converting enzyme 2 (ACE2)	Multiple organs including respiratory tract, heart, liver, brain, kidney, intestine, and other organs express ACE2. It is a critical receptor for SARS-CoV-2.	[23, 101]
CD147 (Basigin or extracellular matrix metalloproteinase inducer)	Megakaryocytes, human bronchial epithelial cells, the brain tissues, and many other organs express CD147. It is an alternative receptor for SARS-CoV-2.	[102, 103]
Tyrosine-protein kinase receptor UFO (ALX)	Pulmonary, marrow stromal cells, bronchial epithelial cells, and most human organs express ALX. It is an alternative receptor for SARS-CoV-2.	[32, 104]
Neuropilin 1 (NRP1)	Olfactory, cerebral area, and respiratory epithelial cells express NRP1. It is an alternative receptor for SARS-CoV-2.	[33, 34]
C-Type Lectins	Dendritic cells, macrophages, endothelial cells of lungs, lives, and renal vessels express C-Type lectins. They are alternative receptor or co-receptor for SARS-CoV-2.	[105, 106]

## Other host factors supporting SARS-CoV-2 infection in human brains

Recent studies showed that lectins and phosphatidylserine receptors could increase viral entry (Table 1), but they did not support efficient SARS-CoV-2 infection in the absence of ACE2 [28]. These factors were named as the attachment factors, like heparan sulfate [29]. CD147 is a transmembrane glycoprotein of immunoglobuline superfamily and has been considered as an alternative receptor for SARS-CoV-2 [30], but the other study showed that it was less likely to be the direct receptor for SARS-CoV-2 infection [31]. The tyrosine-protein kinase receptor UFO (ALX) was also suggested to be a potential receptor for SARS-CoV-2 in pulmonary and bronchial epithelial cells [32]. Apart from these host factors (including co-attachment factors and co-receptors) have been identified for supporting SARS-CoV-2 infection [28], the membrane protein Neuropilin 1 (NRP1) expressed in olfactory, cerebral area, and respiratory epithelial cells was studied as a host factor for SARS-CoV-2 infection in brains [33, 34], which further indicated the possible route of SARS-CoV-2 infection in the brain.

## SARS-CoV-2 infection causing negative effects on nervous systems

Since COVID-19 pandemic emerged in 2019, many patients have reported the long-term symptoms after SARS-CoV-2 infection. These symptoms are now called as Long-COVID syndrome and often include fatigue, predominant neurologic and psychiatric symptoms, such as frequent headaches, shortness of breath, difficulty with memory and concentration, autonomic dysfunction, and other neurologic symptoms [35]. Studies showed that over 60 physical and psychological symptoms were reported to persist up to seven months after SARS-CoV-2 infection [36] and around 10% of adults infected with SARS-CoV-2 appeared to have Long-COVID symptoms [37]. The ACE2, NRP1 and the transmembrane serine protease 2 (TMPRSS2) were detected in human nerves near the medulla [38], which is likely to cause the loss of taste in COVID-19 patients, and other CNS regions [39]. Recently, researchers compared the brain changes before and after SARS-CoV-2 infection by investigating the MRI images of 785 participants [40]. The results showed that overall brain sizes and volume loss were greater in patients with SARS-CoV-2 infection when compared with those in the non-infectious participants. Most of the infectious patients were mild COVID-19 without hospitalization. The results might indicate that even mild COVID-19 can cause the loss of brain volume although there were some limitation in the study, such as 97% participates were aged more than 50 years [41]. The rare detection of SARS-CoV-2 RNA in cerebrospinal fluid from patients [42] suggested that SARS-CoV-2 infection in the brain might not be the major reason for the cognitive problems in some patients. The immune activation and inflammation within CNS might be the primary reasons for neurologic symptoms. However, brain organoid studies had showed that SARS-CoV-2 can replicate with increased viral titers [27] and non-human primate studies showed that SARS-CoV-2 could definitely replicate in the monkey brains [43]. The incidence of parkinsonism was significantly higher in COVID-19 patients when compared to patients infected with influenza virus [6]. Moreover, SARS-CoV-2 infection could activate the Alzheimer’s-like neuropathology [44], which indicated that SARS-CoV-2 infection could cause negative effects on nervous systems [6].

## Effects of SARS-CoV-2-mediated syncytia

**Fig. 2:**
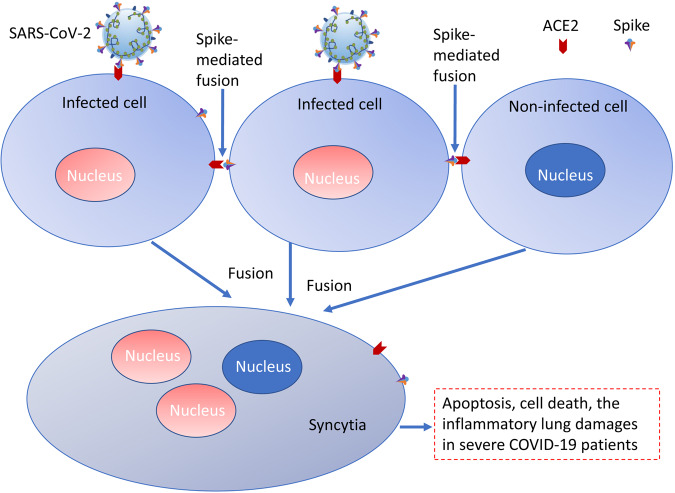
The model of SARS-CoV-2 infection causing syncytia and cell death. Cells infected with SARS-CoV-2 could express spike protein on the cell surface, which can interact with ACE2 on the surface of infected or non-infected cells and then trigger the cell-cell fusion to form syncytia. The syncytia could induce apoptosis and cause inflammatory damages in different organs.

Apart from viral replication, SARS-CoV-2 spike protein could induce cell-cell fusion [45, 46]. Spike-ACE2-mediated fusion could cause multiple syncytia in cells and in the patient lungs [47, 48]. The syncytia formation was suggested to be related to the severe pneumonia and severe immune responses in patients [46]. Apoptosis can be the one possible result of syncytia formation [49]. Theoretically, if only there is ACE2 expression and spike protein expression on two adjacent cell surfaces, these cells can fuse together to form syncytia [Fig. 2]. Virus-induced syncytia is associated with immunosuppression and can enable viruses to evade host defences [50]. Arginine in spike protein of SARS-CoV-2 could affect host Ca^2+^ and transmembrane protein 16F (TMEM16F), which could trigger the syncytia [51–53]. The pathological syncytia tended to internalize lymphocytes to trigger cell-cell fusion and cell death [54]. Various evidences showed the pathological characteristic of syncytia in lungs of COVID-19 patients [51]. In addition, the invasion of SARS-CoV-2 into the brain might cause severe effects on brains, because only spike protein without replicating virus can cause syncytia which could cause apoptosis and cell death [54, 55]. Moreover, human brain organoids and nervon cells expressing spike protein could lead to the formation of syncytia [56]. SARS-CoV-2-indcued fusion was correlated with the neurodegenerative disorders [50]. The syncytia formation in brains might be a potential risk factor for Long-COVID syndrome, which warrants further investigations on the pathogenesis of spike-induced syncytia to nervous systems.

## SARS-CoV-2 infecting the brain regions for memory storage

Memory abilities are the important factors affecting the quality of daily life, human’s academic performance and development, which are considered as the central problems in psychology and social sciences. Most people have not thought much about memory until someday they forget a person’s name or a place name that they used to be familiar with. In the real word, the patients with Alzheimer’s disease demonstrate how important memories are for the simple daily life. SARS-CoV-2 infection was demonstrated in the cognitive center of brain and caused Alzheimer’s like neuropathology [44]. Human memories can be stored across different and interconnected brain regions. They are thought to be initially stored with the hippocampal-entorhinal contex and then slowly moved to the neocortex for permanent storage [57]. Multiple regions are involved in the memory storages, including hippocampus, amygdala, retrosplenial cortex, and prefrontal cortex [58]. For explicit memories (conscious recollection of previous experiences), there are three important brain regions (the hippocampus, the neocortex, and the amygdala) involved. For implicit memories (past experiences that are acquired and retrieved unconsciously), they are relying on the basal ganglia and cerebellum [59]. According to the memory time, the short-term memory or the working memory is mainly stored in the prefrontal cortex. The long-term memory or the long-lasting memory is stored in the hippocampus, amygdala, and the neocortex [59]. Structure changes indicated the reduction in the size of orbitofrontal context, olfactory cortex, and parahippocampal gyrus, which were demonstrated to be associated with SARS-CoV-2 infection [40, 60]. SARS-CoV-2 protein could be stained in cortical neurons and endothelial cells [23] and SARS-CoV-2 RNA could be detected in multiple regions of human brains [61]. Brain tissues from patients showed SARS-CoV-2 replication in astrocytes and consequently it led to neuronal death or dysfunction [62]. The cytokine interleukin-6 (IL-6), tumor necrosis factor-α (TNFα), and interleukine-1β (IL-1β) stimulated by SARS-CoV-2 infection could have a profound impact on working memory and the cognitive systems [63]. The increased expression of IL-6 and TNFα induced by SARS-CoV-2 infection through TLR signaling could cross the blood–brain barrier to activate microglia with the release of IL-1β, which had been demonstrated to interfere with the memory [63]. Overall, memory storage involves multiple regions and can be directly and indirectly affected by SARS-CoV-2 infection. Various symptoms of memory impairments, including short-term memory, working memory, concentration, decision, confusion, problems with daily activities and other related memory problems had been widely reported in COVID-19 patients [63–66].

## SARS-CoV-2 infection affecting children’s memory

The Long-COVID syndrome was not only observed in adults, but also present in children. Various symptoms of the Long-COVID in children had been reported in many cases [65], also including the short-term memory problem after SARS-CoV-2 infection [67]. When compared with symptoms including lack of concentration, difficulty in understanding instruction, and difficulty in processing information [67], the short-term memory problem in children after SARS-CoV-2 infection can be more correlated with the infection. The short-term memory problem will cause the long-term memory problem because it is processed from the short-term memory [57]. These Long-COVID symptoms, especially memory problems, can significantly affect the self-confidence and development of children in coming years. Children under 10–12 years are supposed to be the most important stages for the brain development [67, 68], which implicates that the effects of viral infection on children’s brains can be more destructive. Also, SARS-CoV-2 infection in the central nervous system of a 14-month-old child was reported [69], which demonstrated the viral infection in the region of choroid plexus by both of anti-spike staining and RT-qPCR assay. Moreover, a study showed that children patients with mild COVID-19 could have Long-COVID symptoms with more than 4 months [70]. Studies going to investigate the effects of the Long-COVID syndrome on children’s memory and prevention strategies to reduce the adverse effects are worthwhile.

## The possible mechanisms of SARS-CoV-2 infection affecting memory

Firstly, SARS-CoV-2 can infect brain cells in different brain regions [23], which can be the direct reason of causing the brain damage to affect human memory (Fig. 1). Virus infection activates the immune system through Toll-like receptors, RIG-I-like receptors, NOD-like receptors, and inflammasome sensors to induce cytokine production and cell death which can reduce viral replication and prevent viral spread by eliminating the infected cells [71]. However, hyperactivation of immune responses to viruses can be associated with cytokine storm and severe disease, especially when viruses invading into the brain. In addition, the expression of spike protein of SARS-CoV-2 on cell surfaces can trigger syncytia formation [55]. SARS-CoV-2 infection in brain organoids and human brain tissues can cause cell death [23], which can induce cell apoptosis to trigger multiple immune responses in the brain with long-term effects. Even though no high viral RNA titers were detected in human brains [61], SARS-CoV-2 could effectively replicate in mouse brains when virus was intranasally inoculated to mice which indicated that nasal or lung infection in mice could allow SARS-CoV-2 invading brains [23]. Moreover, SARS-CoV-2 RNA could be detected in non-human primates [43] and SARS-CoV-2 spike protein could be detected in human brains [23]. These results suggested that SARS-CoV-2 invading the brain is possible route to affect human brain and memory.

Secondly, microglial activation and increased cytokine expression (IL-6 and IL-1β) within the hippocampus might explain learning and memory disfunctions in COVID-19 patients [72]. The assay from the brain tissues of COVID-19 patients might mainly represent results in the severe cases at the late stages of infection. For the mild to moderate cases, there was probably no detectable viral RNA in cerebrospinal fluid (CSF), which might be due to the low viral replication in the patient brains [42]. Loss of gray matter within the temporal lobe [40] and reduced memory performance in COVID-19 patients indicated that SARS-CoV-2 infection might increase the risk for later neurodegeneration and dementia [73]. Thus, viral infection outside of the brain triggering the cytokine responses might be the indirect mechanism of dysregulation of the immune system to affect human brain and memory [Fig. 1]. Studies showed that high expression levels of ferritin, C-reactive protein, D-dimer emerge in the blood during SARS-CoV-2 infection [74, 75]. The high production of cytokine IL-6 in the serum of SARS-CoV-2 patients was associated with a negative prognosis [76]. The activation of CD4^+^ and CD8^+^ T cells during SARS-CoV-2 infection and subsequent production of neutralizing antibodies are the key immune responses in humans. The CD4^+^ and CD8^+^ T cells can patrol and protect the border of CNS, while CD8^+^ T cells can provide a cytotoxic defense inhibiting viral infection in the brain [77, 78]. The T cells might contribute to cytokine production during SARS-CoV-2 infection. Recent studies reported that the presence of autoantibodies in patients with the severe courses. About 10% of the tested patients with severe COVID-19 generated antibodies against proteins in blood vessels, hearts, and brains [79, 80]. These findings might explain why there was the delay progress of symptoms after virus was not detectable in some patients. These studies suggested that even without the direct infection of SARS-CoV-2 in the brain, host immune responses against SARS-CoV-2 might also affect the normal function of the brain by cytotoxic defense or autoantibodies.

Thirdly, the persistence of SARS-CoV-2 in immunological sanctuaries could be the potential risk to affect the multiple organs including the brain with long-term effects (Fig. 1). There were reports showing that SARS-CoV-2 could be detected for more than 3 months in patients with severe or mild symptoms [81, 82], patients of young children [83], asymptomatic young adults [84], and patients with immunodeficiency [85]. The persistent SARS-CoV-2 infection in patients might not only cause the persistent stimulation on host immune responses but also increase the possibility of viral transmission and the chance of emerging resistant mutants against vaccines. The immunosuppression and the low ribosomal protein expression were related to the persistent SARS-CoV-2 infection in patients [86]. The persistent SARS-CoV-2 infection in human bodies or brains might provide the long-term signal to stimulate the cytokine expression in the brain, which could affect the function of brain and human memory. Moreover, studies showed that only spike protein without virus could induce neuroinflammatory and behavioral sickness responses [87]. These studies indicated the long-term effects of persistent SARS-CoV-2 infection on human brain and memory.

## Risk factors contributing to Long-COVID syndrome

SARS-CoV-2 infection is the primary reason causing the long-term effects on human brain and memory. Immune dysfunction induced by SARS-CoV-2 infection, the cytokine storm, syncytial formation, and autoantibodies could be the potential factors affecting human brain and memory functions in Long-COVID patients [88]. Other factors, including microclots and biopsychosocial aspects, may also contribute to the Long-COVID disease (Table 2). The extensive fibrin amyloid microclots in individuals with Long-COVID syndrome could entrap other proteins which might induce the production of autoantibodies [89, 90]. The microclots might play roles in affecting the long-term symptoms of SARS-CoV-2 infection. Studies showed the lack of correlation between COVID-19 severity and symptom burden of Long-COVID [91], in which researchers suggested that COVID-19 symptom burden may be related to the psychosocial effects of the COVID-19 pandemic than the infection itself [92]. Recent studies suggested that the Long-COVID patients were more likely to have e severe infections requiring hospitalization [93]. Other studies showed COVID-19 symptoms correlated with the increases in perceived stress and alcohol consumption [94]. The relationship between Long-COVID and biopsychosocial effects and the underline mechanisms of biopsychosocial effects on COVID-19 patients need further studies.

**Table 2:** Risk factors for Long-COVID.

Risk factors	Possible effects on Long-COVID	Reference
SARS-CoV-2 infection	The infection of SARS-CoV-2 can cause cell death and immune dysfunction, which is the direct reason to cause the long-term effects on human organs and brains. Around 10% of people infected with SARS-CoV-2 are estimated to have Long-COVID.	[2, 107]
Cytokine storm	SARS-CoV-2 can activate the immune system through TLR, RLRs, and NRSs signaling to produce cytokines and induce cell death. Dysregulated release of cytokines can conversely cause cytokine storms to lead severe diseases to exacerbate the symptoms of Long-COVID.	[71, 108]
Syncytia	The fusion activity of spike protein of SARS-CoV-2 with host cells can trigger multiple cell fusion in infected organs, which can induce cell death, hyperimmune responses, and the long-term symptoms during the recovery from SARS-CoV-2 infection.	[46, 51]
Autoantibodies	Autoantibodies against type I IFN increase the risk of sever COVID-19 and result in novel type of acquired immunodeficiency, which can prolong the symptoms of Long-COVID.	[90, 109]
Microclots	Microclots can induce autoantibodies in infected organs, which can cause the long-term effects on recovery from SARS-CoV-2 infection.	[90, 110]
Persistent viral infection	It can keep the persistent stimulation on host immune responses and viral protein expression. Spike protein only could induce neuroinflammatory and behavioral sickness responses.	[86, 87]
Biopsychosocial effects	COVID-19 symptoms are associated with emotion stress in some patients, which might be related to the biopsychosocial effects after patients infected with SARS-CoV-2. Biopsychosocial factors might contribute to the fare of COVID-19 and thus affect the recovery from Long-COVID.	[92, 111]

## Prevention strategies for reducing the Long-COVID syndrome

Vaccination is the effective strategy for the prevention of viral infection. SARS-CoV-2 vaccines can reduce the symptoms although current vaccines cannot effectively block SARS-CoV-2 infection. Due to the high transmissibility of SARS-CoV-2 with the increased basic reproduction number (*R*_0_) from the ancestral strain to recent Omicron variants [95], the non-pharmaceutical interventions including wearing masks and keeping distance are also the effective methods to reduce viral transmission and infectious cases. However, the large number of infectious cases are still reported worldwide. Apart from vaccination and non-pharmaceutical controls, new drugs which can limit the viral transmission are urgently needed for containing SARS-CoV-2 transmission and in turn reducing the number of Long-COVID patients.

From the high mortality rates in older population and patients with underling diseases but low mortality rates in young adults, we can suppose that the outcomes of COVID-19 are significantly affected by the immune responses of patients. Especially for the long-term SARS-CoV-2 infection, the low level of immune responses might be the important reason for viral persistent replication without clearance in human bodies [83]. Studies showed that treatment with cytokine-blocking agents in early phases of COVID-19 might show benefits against the later onset of depressive symptoms [96]. Cognitive remediation therapy might reduce long-term cognitive deficits in COVID-19 patients [97]. In addition, good health conditions can help people to keep good status to defend SARS-CoV-2 infection. Frequent exercises, regular sleeping time, and balanced food supply are the three important factors to keep good heathy conditions. The large epidemiologic studies indicated that populations with low death rates showed the common feature of having large quantities of vegetables, including cabbage and fermented vegetables [98]. Moreover, vitamins, zinc, magnesium levels were associated with the severity and deaths of COVID-19 patients [99]. Nutritional supports to provide suitable level vitamins, zinc, and magnesium might reduce inflammation and oxidative stress, which can benefit the human immune systems against SARS-CoV-2 infection. Ecological studies showed the information supporting these factors for preventing SARS-CoV-2 infection with better outcomes [98, 100]. Thus, as the easy way to prevent SARS-CoV-2 infection, good food intake and sleeping time can be the achievable methods for people to establish the natural immune barrier against SARS-CoV-2.

## Discussion

Because of the huge number of SARS-CoV-2 infectious cases, even 10% patients with Long-COVID symptoms can cause heavy burden to human society. The effects of SARS-CoV-2 infection on human brain and memory can affect children and adult’s development and their self-confidence. Current studies related to the effects of SARS-CoV-2 infection on human brain, especially the documented evidences of SARS-CoV-2 affecting human brain and memory have provided the convinced information to raise up the concern of the negative effects of Long-COVID on our daily life. The possible routes of brain damages caused by SARS-CoV-2 infection (including direct viral infection in the brain, immune disfunction, and persistent viral infection) provided the information for better understanding how brain and memory can be affected. There are still largely unknown issues related to the brain damages directly and indirectly caused by SARS-CoV-2 infection. More studies are needed for further understanding Long-COVID pathogenesis. Prevention strategies (including vaccination, antiviral and symptomatic treatment, wearing mask, suitable exercises, regular sleeping time, and balanced food supply) are important in reducing the risk factors of SARS-CoV-2 infection on human brain and memory, especially for children because they are young and their brains are in the early stage of development. Long-COVID symptoms can affect their daily life more destructive with the long-term effects, such as suffering from the loss of memory abilities which can significantly affect their study efficiency and self-confidence before they grow up. Overall, further studies and knowledge about reducing the risks of SARS-CoV-2 infection and preventing SARS-CoV-2 infection can more effectively protect us from SARS-CoV-2 infection.
